# K^+^ efflux through postsynaptic NMDA receptors suppresses local astrocytic glutamate uptake

**DOI:** 10.1002/glia.24150

**Published:** 2022-01-27

**Authors:** Olga Tyurikova, Pei‐Yu Shih, Yulia Dembitskaya, Leonid P. Savtchenko, Thomas J. McHugh, Dmitri A. Rusakov, Alexey Semyanov

**Affiliations:** ^1^ Shemyakin‐Ovchinnikov Institute of Bioorganic Chemistry Russian Academy of Sciences Moscow Russia; ^2^ Department of Clinical and Experimental Epilepsy UCL Institute of Neurology, Queen Square London UK; ^3^ Brain Science Institute (BSI) RIKEN Wako‐shi Saitama Japan; ^4^ RIKEN Center for Brain Science, Wako‐shi Saitama Japan; ^5^ Department of Clinical Pharmacology, Sechenov First Moscow State Medical University Moscow Russia

**Keywords:** astrocyte, depolarization, glutamate uptake, NMDA receptor, potassium, spillover

## Abstract

Glutamatergic transmission prompts K^+^ efflux through postsynaptic NMDA receptors. The ensuing hotspot of extracellular K^+^ elevation depolarizes presynaptic terminal, boosting glutamate release, but whether this also affects glutamate uptake in local astroglia has remained an intriguing question. Here, we find that the pharmacological blockade, or conditional knockout, of postsynaptic NMDA receptors suppresses use‐dependent increase in the amplitude and duration of the astrocytic glutamate transporter current (I_GluT_), whereas blocking astrocytic K^+^ channels prevents the duration increase only. Glutamate spot‐uncaging reveals that astrocyte depolarization, rather than extracellular K^+^ rises per se, is required to reduce the amplitude and duration of I_GluT_. Biophysical simulations confirm that local transient elevations of extracellular K^+^ can inhibit local glutamate uptake in fine astrocytic processes. Optical glutamate sensor imaging and a two‐pathway test relate postsynaptic K^+^ efflux to enhanced extrasynaptic glutamate signaling. Thus, repetitive glutamatergic transmission triggers a feedback loop in which postsynaptic K^+^ efflux can transiently facilitate presynaptic release while reducing local glutamate uptake.

## Main Points

- Potassium efflux through postsynaptic NMDA receptors depolarizes perisynaptic astroglia.
- Potassium‐mediated depolarization reduces astrocytic glutamate uptake.
- Reduced uptake boosts glutamate spillover actions.

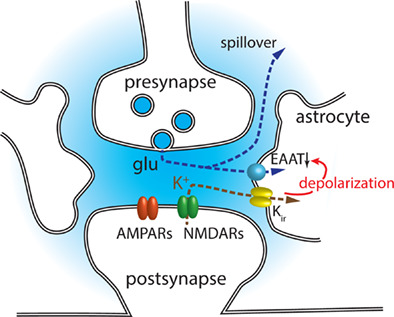

## 1 INTRODUCTION

K^+^ escapes brain cells through K^+^ channels, ion transporters, and ionotropic receptors. Once activated, the main receptors of excitatory glutamatergic synapses, AMPA and NMDA type, are both permeable for K^+^ (Wollmuth & Sobolevsky, 2004). K^+^ efflux through these receptors makes synaptic transmission energetically costlier as it requires more Na^+^ ions entering the cell to generate an excitatory postsynaptic current (EPSC). Nevertheless, K^+^ permeability of AMPA and NMDA receptors (Rs) has been evolutionary conserved, arguably reflecting a signaling role for K^+^. Indeed, transient K^+^ accumulation in the synaptic cleft depolarizes the adjacent presynaptic terminal, which boosts action‐potential‐triggered presynaptic Ca^2+^ entry and therefore increases glutamate release probability (Contini et al., 2017; Hori & Takahashi, 2009). Patch‐clamp experiments combined with biophysical simulations have suggested that, during synaptic transmission, the bulk of intra‐cleft K^+^ arrive through postsynaptic NMDARs that remain activated for 100–200 ms, rather than through short‐lived K^+^ efflux due to AMPAR activation or during presynaptic spike repolarization (Shih et al., 2013). This retrograde signaling also depends on postsynaptic depolarization which is required to remove the Mg^2+^ block of NMDAR channels (Nowak et al., 1984). The extracellular K^+^ elevation generated during synaptic transmission is cleared by diffusion, neuronal and astrocytic Na^+^/K^+^ ATPase, astrocytic inward‐rectifying K^+^ channels (K_ir_), and various other channels and transporters. K^+^ current through astrocytic K_ir_ channels could last for hundreds of milliseconds in response to a single synaptic stimulus, suggesting a relatively long dwell time of extracellular K^+^ transients (Cheung et al., 2015; Lebedeva et al., 2018; Meeks & Mennerick, 2007). Because the membrane potential of astrocytes is predominantly determined by their K^+^ conductance, increases in extracellular K^+^ could be sufficient to depolarize local astrocytic membranes. Although the latter should, in theory, reduce voltage‐dependent glutamate uptake through the main glial glutamate transporter GLT‐1 (Grewer et al., 2008; Grewer & Rauen, 2005; Mennerick et al., 1999), whether this sequence of events takes place in reality remains largely unknown. Intriguingly, induction of synaptic long‐term potentiation has been shown to boost extrasynaptic glutamate escape, by driving withdrawal of local astrocytic processes (Henneberger et al., 2020). However, it has remained unknown if any similar effect occurs in the course of regular excitatory activity. Because a better understanding of such phenomena should shed light on the fundamental rules of local signal integration in excitatory brain circuits, we have embarked on a multi‐disciplinary study to explore them.

## 2 MATERIALS AND METHODS

### 2.1 Hippocampal slice preparation

Animal procedures were carried out under the oversight of the UK Home Office (as per the European Commission Directive 86/609/EEC and the UK Animals [Scientific Procedures] Act, 1986) and by institutional guidelines. Young C57BL/six mice (3–4 weeks of age) male were anesthetized using isoflurane and decapitated (at the time of virus injection, we could not identify the animal's sex with certainty, and later we were compelled to use all injected animals, in accord with the 3Rs principles, the bulk of which were males). The brain was exposed, chilled with an ice‐cold solution containing (in mM): sucrose 75, NaCl 87, KCl 2.5, CaCl_2_ 0.5, NaH_2_PO_4_ 1.25, MgCl_2_ 7, NaHCO_3_ 25, and D‐glucose 25. Hippocampi from both hemispheres were isolated and placed in an agar block. Transverse slices (350 μm) were cut with a vibrating microtome (LeicaVT1200S) and left to recover for 20 min in the same solution at 34°C. Then slices were incubated at 34°C in a solution containing (in mM): NaCl 119, KCl 2.5, NaH_2_PO_4_ 1.25, MgSO_4_ 1.3, CaCl_2_ 2.5, NaHCO_3_ 25, and D‐glucose 11. For experiments with intracellular blockade of K_ir_, the solution was supplemented with 100 μM BaCl_2_. After that, slices were transferred to the recording chamber and continuously perfused at 34°C with the same solution. All solutions were saturated with 95% O2 and 5% CO2. Osmolarity was adjusted to 298 ± 3 mOsM.

### 2.2 AAV transduction

For viral gene delivery of AAV2/1 h.Synap.SF‐iGluSnFR‐A184V (Penn Vector Core, PA, USA), pups, male and female (P0‐P1), were prepared for aseptic surgery. To ensure proper delivery, intracerebroventricular (ICV) injections were carried out after an adequate visualization of the targeted area (Kim et al., 2014), as described previously (Kopach et al., 2020). Viral particles were injected in a volume 2.5 μL/hemisphere (totaling 5 × 10^9^ genomic copies), using a glass Hamilton microsyringe at a rate not exceeding of 0.2 μL/s, 2 mm deep, perpendicular to the skull surface, guided to a location approximately 0.25 mm lateral to the sagittal suture and 0.50–0.75 mm rostral to the neonatal coronary suture. Once delivery was completed, the microsyringe was left in place for 20–30 s before being retracted. Pups (while away from mothers) were continuously maintained in a warm environment to eliminate the risk of hypothermia in neonates. After animals received AAV injections, they were returned to the mother in their home cage. Pups were systematically kept as a group of litters. Every animal was closely monitored for signs of hypothermia following the procedure and for days thereafter to ensure that no detrimental side effects appear. For the transduction of glutamate sensors in vivo, there were three‐ to four‐ weeks to suffice.

### 2.3 Electrophysiology

**FIGURE 1:**
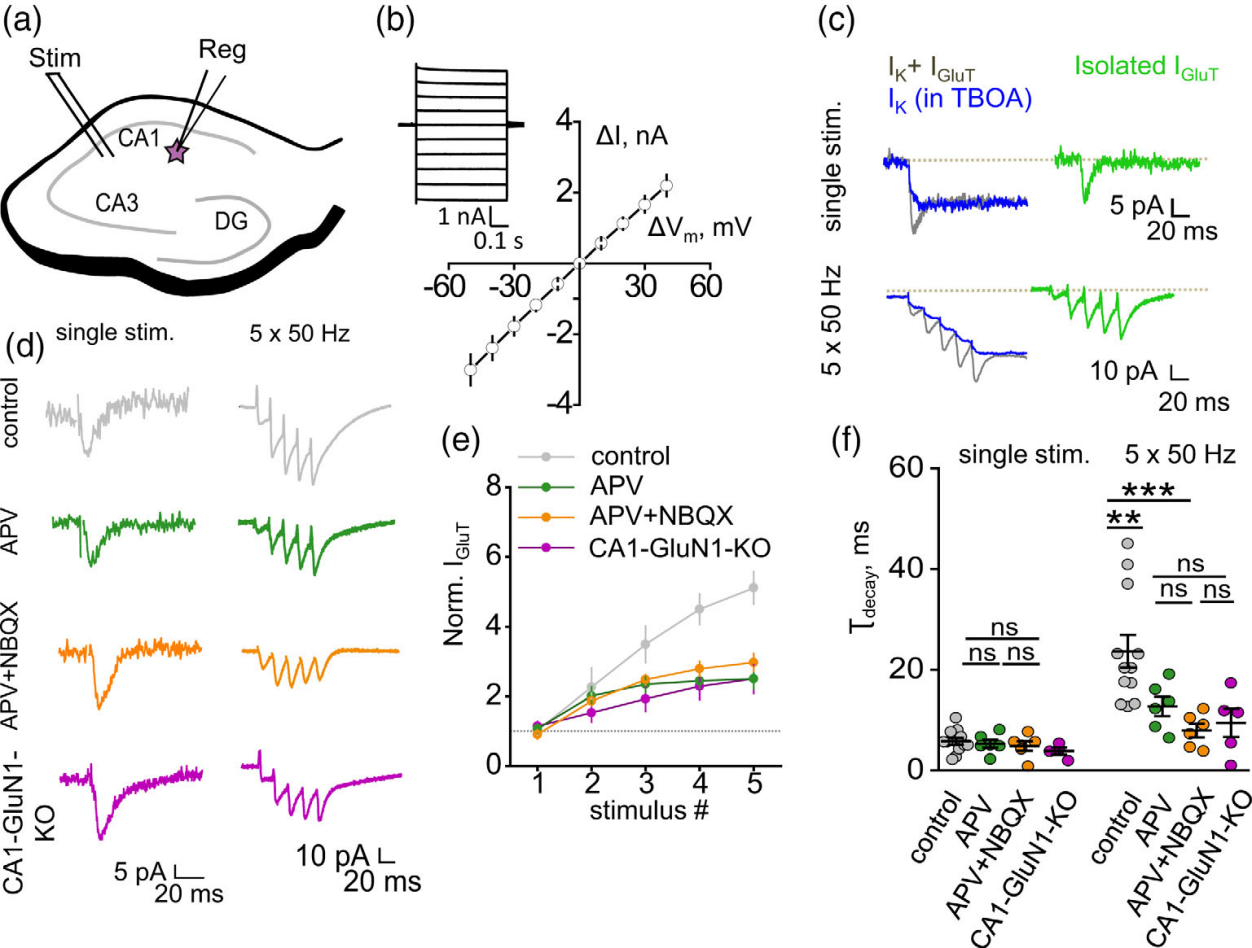
NMDAR‐mediated K^+^ efflux induces an activity‐dependent increase of I_GluT_ amplitude and decay time. (a) A schematic showing stimulating (SC stim) and recording (Reg) electrode positions for the recording of synaptically‐induced currents in CA1 *stratum radiatum* astrocyte. (b) Current–voltage relationship of passive astrocyte. *Inset*: Astrocytic current (ΔI) was measured in response to voltage steps (ΔV_m_). (c) Astrocytic currents induced by single and 5 × 50 Hz stimulation of Schaffer collaterals. DL‐TBOA application was used to isolate I_K_ (blue), which was then subtracted from combined current (I_K_ + I_GluT_, gray) to obtain I_GluT_ (green). (d) Sample traces of I_GluT_ in response to single and 5 × 50 Hz stimulation in control (gray), in the presence of D‐APV (green), in the presence of D‐APV (orange) and recorded from CA1‐GluN1‐KO mice (purple). (e) Amplitudes of I_GluT_ peaks during 5 × 50 Hz stimulation normalized to the amplitude of I_GluT_ in response to a single stimulus. Activity‐dependent facilitation in control (gray) was reduced by the application of D‐APV (green) or D‐APV + NBQX (orange) or in CA1‐GluN1‐KO mice (purple). (f) τ_decay_ of I_GluT_ in response to single and 5 × 50 Hz stimulation. There was no significant difference in τ_decay_ of I_GluT_ in response to a single stimulus between control (gray), D‐APV (green), D‐APV + NBQX (orange) or in CA1‐GluN1‐KO mice (purple). τ_decay_ of I_GluT_ in response 5 × 50 Hz stimulation was significantly larger in control than in D‐APV, D‐APV + NBQX, or in CA1‐GluN1‐KO mice. The data are presented as mean ± SEM. ns *p* > .05, **p < .01 and ***p < .001, two‐sample *t* test

Whole‐cell recordings from *stratum radiatum* astrocytes were obtained using patch electrodes filled with a potassium methane sulfonate‐based solution (KMS) containing (in mM): CH_3_KO_3_S 135, HEPES 10, MgCl_2_ 4, disodium phosphocreatine 10, Na_2_ATP 4, NaGTP 0.4 (pH adjusted to 7.2 with KOH; osmolarity to 290 ± 3 mOsM) with a resistance of 3–5 MΩ. The 50 μM Alexa Fluor 594 was added to the intracellular solution as a morphological marker. Astrocytes were identified by small soma size (about 10 μm), low resting membrane potential (−84.0 ± 0.5 mV, *n =* 16), and low input resistance (16.3 ± 1.4 MΩ, *n =* 16). Passive cell properties were confirmed by linear I–V characteristics (Figure 1b). Synaptic responses were evoked by single and burst stimulation (5 stimuli at 50 Hz) of Schaffer Collaterals (SC) with a bipolar electrode (FHC, Bowdoin, USA). The stimulating electrode was placed in the *stratum radiatum* more than 200 μm from the recording site. Signals were amplified with the Multiclamp 700B amplifier (Molecular Devices), filtered at 2 kHz, recorded and digitized at 4–10 kHz with the NI PCI‐6221 card (National Instruments).

Whole‐cell recordings of CA1 pyramidal neurons were obtained using patch electrodes filled with a solution containing (in mM): CH_3_KO_3_S 130, NaCl 8, HEPES 10, disodium phosphocreatine 10, Na_2_GTP 0.4, MgATP 4, and 3 mM Na‐ascorbate (pH adjusted to 7.2 with KOH; osmolarity to 290 ± 3 mOsM). To eliminate postsynaptic K^+^ efflux, CH_3_KO_3_S was replaced with N‐methyl‐D‐glucamine CH_3_SO_3_ (NMDG).

### 2.4 Glutamate uncaging

The 4‐methoxy‐7‐nitroindolinyl‐caged L‐glutamate (10 mM, MNI‐glutamate) was applied to the perfusion solution. Glutamate uncaging was carried out using mode‐locked tunable 690–1020 nm laser Mai‐Tai (Spectra‐Physics, USA) in a “point scan” mode for 5 ms at 720 nm with the FV1000‐MPE system, as described previously (Henneberger et al., 2020; Savtchenko et al., 2018).

### 2.5 iGluSnFR imaging

Femtonics Femto2D‐FLIM imaging system was used for two‐photon imaging, integrated with two femtosecond pulse lasers MaiTai (SpectraPhysics‐Newport) with independent shutter and intensity control and patch‐clamp electrophysiology system (Femtonics, Budapest). Patch pipettes were pulled from borosilicate–standard wall filament glass (G150F‐4; Warner Instruments, CT, USA) with 4–5 MΩ resistance. CA1 pyramidal neurons expressing iGluSnFR sensor were patch‐clamped with either KMS‐ or NMDG‐based internal solution, supplemented with the morphological tracer dye Alexa Fluor 594 (50 μM). The Alexa Fluor 594 channel was used to identify a region of interest and recorded along with the iGluSnFR signal. After at least 45 min required for the dye diffusion and equilibration in the dendritic arbor, glutamate imaging from individual spines was carried out using an adaptation of the previously described method (Henneberger et al., 2020; Jensen et al., 2017). For the fast imaging of AP‐mediated glutamate transients point scans were performed over the dendritic spines and scanned at a sampling frequency of 500 Hz.

### 2.6 Astrocyte simulations

Simulations were carried out using a detailed 3D biophysical model of a (statistically) reconstructed CA1 astrocyte using a NEURON‐compatible model builder ASTRO (www.neuroalgebra.com and https://github.com/LeonidSavtchenko/Astro), as outlined in detail earlier (Savtchenko et al., 2018). In brief, ‘average’ astrocyte morphology was obtained from a sample of protoplasmic astrocyte in hippocampal *stratum radiatum* (~4‐week‐old rats) using the systematic procedures as follows. First, generating soma and primary (optically discernible) branches to match sample‐average numbers and dimensions; second, adding nanoscopic branches that have statistically generated dimensions matching experimental EM data (size distributions); third, adjusting biophysical properties of nano‐branches to match biophysics of reconstructed 3D EM processes; fourth, adjusting the numbers and density of nano‐branches to match the statistics of tissue volume fraction and surface‐to‐volume ratios obtained empirically from 3D EM data; fifth, distributing biophysical membrane mechanisms across the model cell membrane, including K_ir_4.1 channels and GLT‐1 transporters in accord with the experimental recordings. A full description of the model and its implementation, for either desktop‐ or cloud‐computing, are available from www.neuralgebra.com and links therein.

The model was populated with K_ir_4.1 channel, with the kinetics in accord with (Jérémie Sibille et al., 2015), and membrane unit conductance of 𝐺𝐾𝑖𝑟 =0.1 mS cm^−2^.

The kinetic is described by the equation:

$$I_{\mathrm{Kir}}=G_{\mathrm{Kir}}\left(V-\mathrm{NK}\times E_{k}-V_{A1}\right)\frac{\sqrt{{\left[K^{+}\right]}_{o}}}{1+\mathrm{Exp}\left(\frac{V-\mathrm{NK}\times E_{k}-V_{A2}}{V_{A3}}\right)},$$

where *V*_*A1*_ = −14.83 mV, *V*_*A2*_ = −105.82 mV, *V*_*A3*_ = 19.23 mV, *NK* = 0.81, *E*_*k*_ is the Nernst astrocyte K^+^ potential, *V* is the astrocyte membrane potential, [*K* ^*+*^]_*0*_ is the extracellular *K* ^*+*^ concentration and *V*_*A1*_ (an equilibrium parameter, which sets I_Kir_ current to 0 at −80 mV), *NK,V*_*A2*_ and *V*_*A3*_ are constant parameters calibrated by the I‐V curve.

The leak passive current *I*_*pas*_ *= G*_*pas*_ *(V − E*_*pas*_ *)* was added to stabilize the astrocyte membrane potential at *E*_*pas*_ = −85 mV, *G*_*pas*_ = 0.001 mS/cm^2^.

GLT‐1 kinetics was modeled in accord with (Bergles et al., 2002; Z. Zhang et al., 2007), with the surface density of 10^4^ μm^−2^ as estimated earlier (Lehre & Danbolt, 1998).

The kinetics of glutamate transporters was determined by a simplified scheme of 6 states:

$$\begin{array}{ccccc} C_{1} & \overset{C_{12}}{\underset{C_{21}}{⇄}} & C_{2} & \overset{C_{23}}{\underset{C32}{⇄}} & C_{3}\\ C_{16}↓↑C_{61} & & & & C_{34}↓↑C_{43}\\ C_{6} & \overset{C_{65}}{\underset{C_{56}}{⇄}} & C_{5} & \overset{C_{54}}{\underset{C_{45}}{⇄}} & C_{4} \end{array},$$

where C_12_ = [Glu]_o_ k_12_ u(v,‐0.1), C_21_ = k_21_, C_23_ = [Na^+^]_o_ k_23_ u(v,0.5), C_32_ = k_32_, C_34_ = k_34_ u(V,0.4), C_43_ = k_43_, C_45_ = k_45_, C_54_ = k_54_ [Glu]_in_, C_56_ = k_56_ u(v,0.6), C_65_ = k_65_ [Na^+^]_in_, C_61_ = [K^+^]_in_ k_61_, C_16_ = k_16_ u(V,0.6) [K^+^]_o_.

And function u(V,P) = exp(P V/53.4), where V is astrocyte membrane voltage, and P is a charge translation between states C_i_ ‐ > C_j_.

The value of kinetic constant were: k_12_ = 20 /mM /ms, k_21_ = 0.1 /ms, k_23_ = 0.015 /mM /ms, k_32_ = 0.5 /ms, k34 = 0.2 /ms, k_43_ = 0.6 /ms, k_45_ = 4 /ms, k_54_ = 10 /mM/ms, k_56_ = 1 /ms, k_65_ = 0.1/mM/ms, k_16_ = 0.0016 /mM/ms, k_61_ = 2 10^−4^ /mM /ms.

The glutamate transporter current (Z. Zhang et al., 2007) was calculated according to the following equation:

$$\begin{array}{lll} I_{\mathrm{Glu}} & = & -e\;\mathrm{den}(0.6\left(C_{1}k_{16}{\left[K^{+}\right]}_{o}u\left(V,0.6\right)-C_{6}k_{61}{\left[K^{+}\right]}_{\text{in}}\right)\\ & & -0.1\left(C_{1}k_{12}{\left[\mathrm{Glu}\right]}_{o}u\left(V,-0.1\right)-C_{2}k_{21}\right)+0.5\\ & & \times \left(C_{2}k_{23}{\left[{\mathrm{Na}}^{+}\right]}_{o}u\left(V,0.5\right)-C_{3}k_{32}\right)+0.4\left(C_{3}k_{34}-C_{4}k_{43}\right)\\ & & +0.6\left(C_{5}k_{56}u\left(V,0.6\right)-C_{6}k_{65}{\left[{\mathrm{Na}}^{+}\right]}_{\text{in}}\right)), \end{array}$$

where e is charge on an electron charge 1.6 × 10^−19^ (coulombs) and den is a density of transporters

Initial ion concentrations:

$${\left[{\mathrm{Na}}^{+}\right]}_{\text{in}}=15\;\mathrm{mM},{\left[{\mathrm{Na}}^{+}\right]}_{o}=150\;\mathrm{mM},{\left[K^{+}\right]}_{\text{in}}=120\;\mathrm{mM},{\left[K^{+}\right]}_{o}=3\;\mathrm{mM},{\left[\mathrm{Glu}\right]}_{\text{in}}=0.3\;\mathrm{mM},$$

Diffusion of intracellular potassium is described by the equation built into the Neuron:

~ ka < < (‐I_Kir4.1_/[Fπd]), where F = 96,485 C/M is Faraday constant, d is a local diameter with potassium diffusion coefficient D_K_ = 0.6 mm/ms^2^.

To fit the experimental observation, we fit the kinetic scheme and modified the sensitivity of transporter to the change of voltage when binding [K]o, C16 = k16 u(V,0.6) [K+]o from 0.6 to 0.1.

Extracellular [K^+^]_o_ elevation from 2.5 up to 5 mM was simulated across a 20 μm spherical area within the astrocyte arbor: while diffusing away, it also activated Kir4.1 homogeneously within the area, prompting K^+^ entry into the astrocyte (peak current density ~ 0.01 mA cm^−2^). The ensuing local increase in intracellular K^+^ concentration (from 110 to ~110.5 mM) dissipated over several seconds after extracellular K^+^ concentration returned to the baseline value of 2.5 mM. Extracellular glutamate rise (peak 0.1 mM, 2 ms) was simulated within a 3 μm sphere inside the astrocyte arbor.

The extracellular concentration of glutamate was calculated as a conditions IGlu = 0 at [K]o = 5 [Glu]o = 8.5*10^−5^ mM and [K] = 2.5 mM [Glu]o = 3.57*10^−6^ mM.

### 2.7 Drugs and chemicals

All drugs were made from stock solutions kept frozen at −20°C in 100–200 ml 1000 × aliquots. DL‐2‐amino‐5‐phosphonovaleric acid (D‐APV), 2,3‐dioxo‐6‐nitro‐7‐sulfamoyl‐benzo[f]quinoxaline (NBQX), DL‐threo‐β‐benzyloxyaspartic acid (DL‐TBOA), [5R,10S]‐[+]‐5‐methyl‐10,11‐dihydro‐5H‐dibenzo[a,d]cyclohepten‐5,10‐imine (MK‐801), picrotoxin, and MNI‐ glutamate were purchased from Tocris Cookson (Bristol, UK). Chemicals for solutions were from Sigma‐Aldrich (St. Louis, USA). Alexa Fluor 594 was obtained from Invitrogen (Carlsbad, USA).

### 2.8 Statistical analysis

Electrophysiological data were analyzed with WinWCP and Clampfit (Axon Instruments Inc.; Union City, USA). Imaging data were analyzed using MES software (Femtonics, Budapest), ImageJ (a public domain Java image processing program by Wayne Rasband), and traces expressed as ΔF/F. Statistical analysis was performed using Excel (Microsoft, US), Origin 8 (Origin Lab Corp, Northampton, USA). All data are presented as the mean ± SEM, and the statistical difference between means was estimated with the unpaired *t*‐test and repeated measurements two‐way ANOVA as stated in the text.

## 3 RESULTS

### 3.1 Activity‐dependent increase of I_GluT_ amplitude and the decay time is mediated by postsynaptic NMDARs

Whole‐cell recordings were performed in CA1 *stratum radiatum* astrocytes in acute hippocampal slices of the mouse (Figure 1a). Astrocytes were visually identified by their shape and the linear I‐V curve (Figure 1b). A combined transporter (I_GluT_) and K^+^ (I_K_) current was evoked by extracellular stimulation of Schaffer collaterals (Figure 1c). At the end of each experiment, 50 μM DL‐TBOA, a blocker of glutamate transporters, was added to isolate I_K_. The tail of I_K_ in the presence of DL‐TBOA was scaled to the tail of each individual combined current obtained in response to equivalent stimulation. The scaled I_K_ was subtracted from each corresponding trace of the combined current to obtain the time course of isolated I_GluT_ (Figure 1c).

Earlier work has suggested that NMDAR‐mediated K^+^ efflux produces activity‐dependent presynaptic depolarization and facilitation of glutamate release in synapses on CA1 pyramidal neurons (Shih et al., 2013). Indeed, we observed progressive facilitation of the I_GluT_ amplitude with 5 × 50 Hz stimulation; the facilitation was significantly reduced by 50 μM D‐APV, an NMDARs antagonist (*p*_APV_ < .001; *p*_stimulus_ < .001; interaction: *F*[4, 50] = 3.6, *p* = .01; *n =* 6; two‐way RM ANOVA; Figure 1d, e). The residual I_GluT_ facilitation can be attributed to an increase in presynaptic release probability due to mechanisms independent of the NMDARs‐mediated K^+^ efflux, such as the accumulation of residual Ca^2+^ in the presynaptic terminal. Application of 25 μM NBQX, an AMPAR antagonist, in addition to D‐APV, did not increase the effect, suggesting a minor role for AMPARs in the regulation of glutamate release. Activity‐dependent facilitation of I_GluT_ in the mice with conditional knockout of the GluN1 subunit of NMDARs in CA1 pyramidal neurons (CA1‐GluN1‐KO) (McHugh et al., 1996; Wu et al., 2012) was similar to the effect of D‐APV, confirming the involvement of postsynaptic NMDARs.

The decay time constant (τ_decay_) of I_GluT_ recorded in response to a single stimulus was not significantly affected by D‐APV or by co‐application of D‐APV and NBQX (control: 5.75 ± 0.65 ms, *n =* 12; D‐APV: 5.26 ± 0.80 ms, *n =* 6; *p =* 0.64 for the difference between control and D‐APV; D‐APV + NBQX: 4.83 ± 0.92 ms, *n =* 6; *p =* 0.72 for the difference between control and D‐APV + NBQX; two‐sample *t*‐test; Figure 1f). The τ_decay_ of I_GluT_ recorded in response to a single stimulus in CA1‐GluN1‐KO mice was also not significantly different from τ_decay_ in control mice (KO mice: 3.82 ± 0.69 ms, *n =* 4; *p =* .07 for the difference with control mice; two‐sample *t*‐test; Figure 1f).

The values of τ_decay_ became significantly larger in response to 5 × 50 Hz stimulation (23.65 ± 3.22 ms, *n =* 12; *p <* .001 for the difference with single stimulus; paired‐sample *t*‐test; Figure 1f). This increase was significantly reduced by bath application of D‐APV (τ_decay_ = 12.71 ± 1.91 ms, *n =* 6, *p =* .01 for the difference with control, two‐sample *t*‐test; Figure 1f). Adding NBQX to D‐APV produced a further reduction in τ_decay_, albeit statistically insignificant (τ_decay_ = 7.91 ± 1.35 ms, *n =* 6, *p <* .001 compared to control, *p =* 0.13 compared to D‐APV, two‐sample *t*‐test; Figure 1e). These results are consistent with an earlier suggestion that synaptically released glutamate does not overwhelm glutamate transporters upon the blockade of AMPARs and NMDARs (Diamond & Jahr, 2000). τ_decay_ in CA1‐GluN1‐KO mice was not significantly different from τ_decay_ in D‐APV (KO mice: 9.42 ± 2.82 ms, *n =* 5; *p =* 0.21 for the difference with D‐APV, two‐sample *t*‐test; Figure 1f). These findings suggest that postsynaptic NMDARs are required both for the activity‐dependent facilitation of glutamate release and for the activity‐dependent reduction of glutamate uptake.

### 3.2 Activity‐dependent increase of I_GluT_ decay time does not depend on afferent recruitment

**FIGURE 2:**
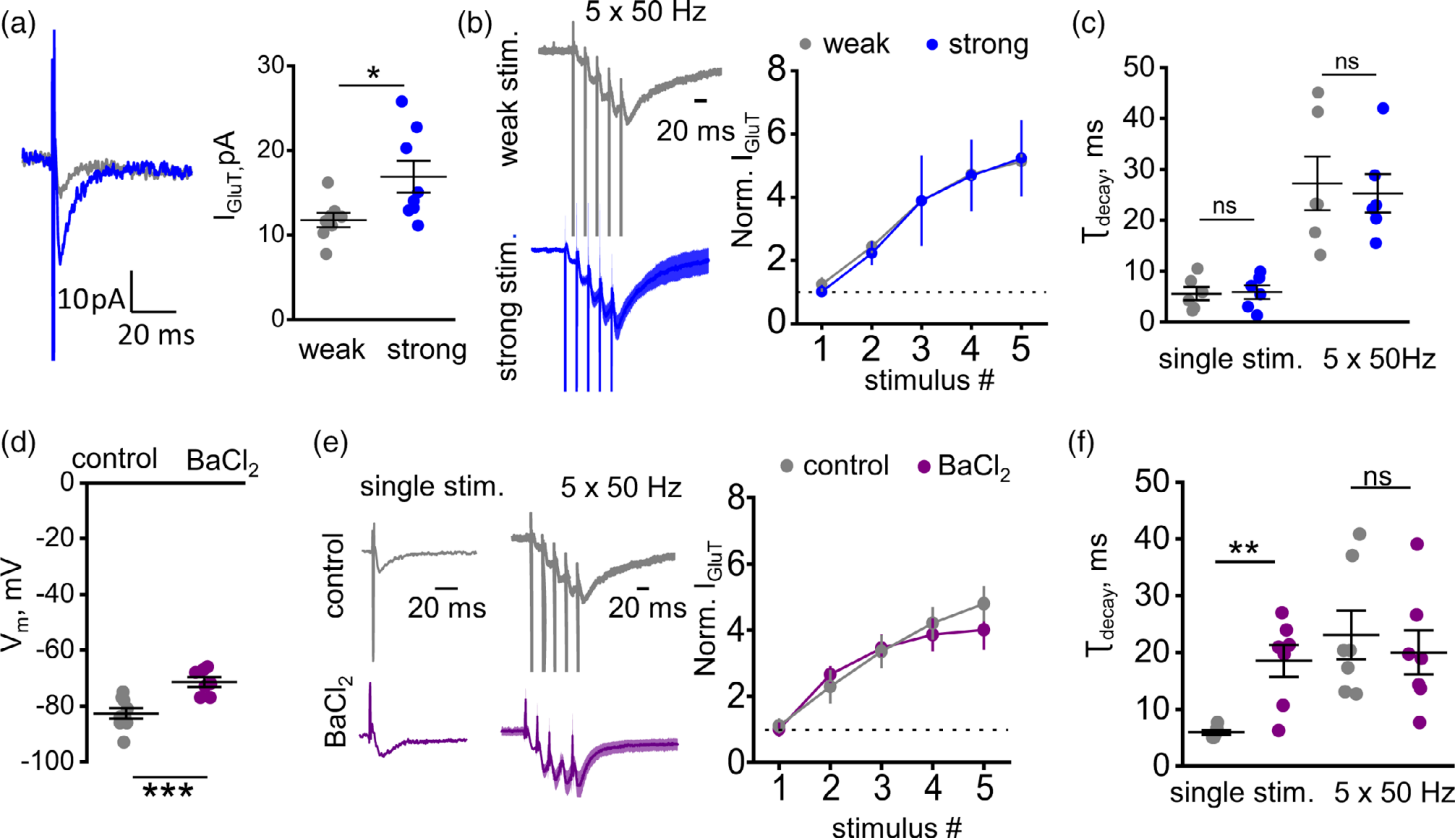
Activity‐dependent increase of I_GluT_ decay time does not depend on the stimulus strength but abolished by the blockade of K_ir_. (a) *Left*: Traces of I_GluT_ recorded with weak (gray) and strong (weak*2; blue) single stimulation of Shaffer collaterals. *Right*: The summary plot showing an increase in the amplitude of I_GluT_ by increasing the stimulation strength twice (from weak to strong).(b) The increase in stimulation strength did not affect the activity‐dependent facilitation of I_GluT_. *Left*: The traces of I_GluT_ in response to 5 × 50 Hz stimulation normalized to the amplitude of I_GluT_ in response to single weak stimulation. The traces were averaged in all experiments and presented as mean ± SEM. *Right*: The summary plot of normalized I_GluT_ amplitude in response to each stimulus in the train. (c) τ_decay_ of I_GluT_ in response to single and 5 × 50 Hz stimulation was not affected by the increase in stimulus strength. Gray circles–weak stimulation; blue circles–strong stimulation. (d) The summary graph showing the effect of BaCl_2_ astrocyte membrane potential. (e) *Left*: Normalized traces of I_GluT_ recorded with control intracellular solution (gray) and the solution containing K_ir_ channels blocker ‐ BaCl_2_ (purple). The traces for 5 × 50 Hz stimulation were averaged in all experiments and presented as mean ± SEM. *Right*: The summary plot of normalized I_GluT_ amplitude in response to each stimulus in the train. (f) The summary graph showing the effect of BaCl_2_ on τ_decay_ of I_GluT_ in response to single and 5 × 50 Hz stimulation. Gray circles–control; purple circles–BaCl_2_. The data are presented as mean ± SEM. ns *p* > .05, *p < .05 and **p < .01, paired‐sample (a, d) and two‐sample (c, f) *t* test

NMDARs activation requires voltage‐dependent unblocking of the receptor channel (Nowak et al., 1984). Therefore, greater postsynaptic depolarization should, in theory, recruit more NMDARs and thus produce larger K^+^ efflux. To test this hypothesis, we recorded I_GluT_s at two different stimulation strengths (Figure 2a). Initially, the stimulation strength was adjusted to achieve I_GluT_ ~ 10 pA (weak stimulus), then the strength was doubled (strong stimulus). Strong stimulation increased the I_GluT_ to 17.07 ± 1.8 pA (*n =* 8; *p =* .03, paired sample *t*‐test; Figure 2a). Surprisingly, we did not observe a significant enhancement in the activity‐dependent facilitation of I_GluT_ upon stronger stimulation (*p*_strengh_ = 0.41; *p*_stimulus_ < .001; interaction: *F*[4, 60] = 0.01, *p* = 0.99; *n =* 7; two‐way RM ANOVA; Figure 2b). Nor did we observe a significant difference in τ_decay_ between weak and strong stimulation groups, either with one or with five stimuli tests (single stimulus: 5.57 ± 1.3 ms for weak stimulation, 5.89 ± 1.34 ms for strong stimulation, *p =* 0.87, paired sample *t*‐test; 5 stims: 27.24 ± 5.29 ms for weak stimulation, 25.29 ± 3.77 ms for strong stimulation, *p =* 0.78, paired sample *t*‐test; *n =* 6; Figure 2c). Thus, although stronger stimulation releases more glutamate per tissue volume, hence generates larger uptake currents, the current kinetics appears unaffected. This is likely because glutamate transporter binding and uptake occur only inside the microscopic vicinity of individual synapses, within 5–10 ms postrelease. Unless such ‘uptake hotspots’ substantially overlap in the tissue volume, engaging additional synapses would not be expected to affect glutamate uptake kinetics.

To provide further controls, we have carried out experiments in which astrocytic K^+^ currents (I_K_) were compared directly under strong and weak stimuli, in conditions of single and burst stimuli. As expected, I_K_ increased with the increased stimulus strength or number. Importantly, in response to a stimulus burst, the use‐dependent increase in I_K_ was similar under either weak or strong stimuli (Figure S1), thus arguing that it scales linearly independently of the number of synapses/afferent fibers involved.

### 3.3 Activity‐dependent increase of I_GluT_ decay time is abolished by the blockade of astrocytic K_ir_

Next, we asked if the activity‐dependent increase in τ_decay_ involves the K_ir_‐dependent depolarization of astrocytic leaflets. Earlier work has shown that dialising astrocytes with 100 μM BaCl_2_ blocks K_ir_ channels responsible for I_K_ in astrocytes (Afzalov et al., 2013). Although the genetic deletion or downregulation of the main astrocytic K_ir_ channel, Kir4.1, could provide useful insights in their functional roles (Sibille et al., 2014), it could also cause sustained membrane depolarization (Djukic et al., 2007), which would in turn suppress the other powerful astrocytic K^+^ clearance mechanism, the Na/K‐ATPase (Larsen et al., 2014). This pump is also sensitive to internal astrocytic Na^+^ which homeostasis might be affected in the Kir4.1‐downregulated cells (Kirischuk et al., 2012). We therefore opted for pharmacological manipulation that would permit real‐time comparison between control and affected cells.

Here, the classical K_ir_ channel blocker BaCl_2_ produced a small but significant depolarization of the recorded astroglia (V_m_ = −82.66 ± 1.85 in control, *n =* 9; V_m_ = −71.42 ± 1.73 in BaCl_2_, *n =* 7; *p <* .001 two‐sample *t*‐test; Figure 2d). Surprisingly, BaCl_2_ had no significant effect on I_GluT_ facilitation (*p*_BaCl2_ = 0.26; *p*_stimulus_ < .001; interaction: *F*[4, 50] = 1.1, *p* = 0.34; *n = 8 for control, n = 6 for BaCl*_*2*_.; two‐way RM ANOVA; Figure 2e). This likely explanation is that K^+^ dynamics in the synaptic cleft depends mainly on diffusion escape rather than on the K_ir_‐mediated clearance (Meeks & Mennerick, 2007). On the other hand, BaCl_2_ significantly increased τ_decay_ in response to a single stimulus (5.93 ± 0.41 ms in control, *n =* 7; 18.35 ± 3.3 ms in BaCl_2_, *n* = 5; *p =* .01, two‐sample *t*‐test; Figure 2f). This finding is consistent with the reduction of electrogenic glutamate uptake following astrocyte depolarization that occurs in perisynaptic astrocytic leaflets upon blockade of K_ir_. Indeed, astrocytes have very leaky membranes in which voltage clamp can be easily disturbed by local currents (e.g., (Savtchenko et al., 2018)). No further activity‐dependent prolongation in τ_decay_ were observed in the presence of BaCl_2_ (τ_decay_ in response to 5 × 50 Hz stimulation: 20.03 ± 4.59 ms, *n =* 7, *p =* 0.6, paired‐sample *t*‐test for difference with single stimulus; Figure 2f). Notably, τ_decay_ in these tests was not significantly different from τ_decay_ in response to 5 × 50 Hz stimulation in control conditions (23.55 ± 5.02 ms in control, *n* = 7; *p =* 0.6, two‐sample *t*‐test for difference with BaCl_2_; Figure 2f).

### 3.4 Astrocyte depolarization but not extracellular K^+^ affects I_GluT_

**FIGURE 3:**
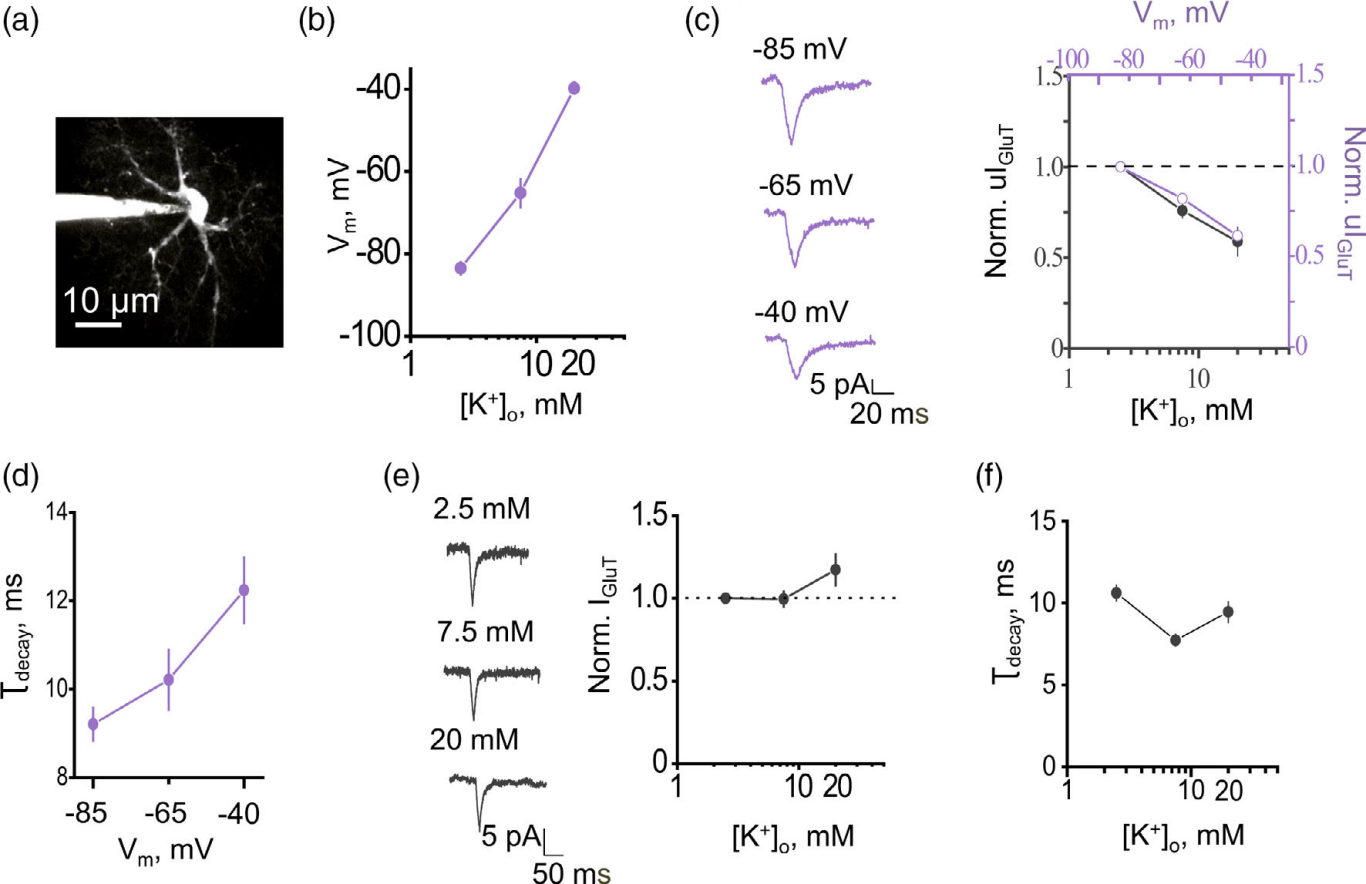
Depolarization of astrocyte rather than a decrease in K^+^ gradient suppresses glutamate uptake. (a) An image of astrocyte filled with Alexa Fluor 594. An uncaging spot was in proximity to soma to ensure proper voltage clamp. (b) Astrocyte depolarization produced by an increase in [K^+^]_o_. (c) *Left*, sample traces of uI_GluT_ produced by glutamate uncaging while holding astrocyte at different membrane potential (V_m_). *Right*, the summary plot showing normalized uI_GluT_ amplitude in the cells voltage‐clamped at −85, −65, and − 40 mV (purple circles) and in the cells clamped at the same voltages but with [K^+^]_o_ corresponding to each voltage as in (b) (2.5, 7.5, and 20 mM; black circles). K^+^ elevation did not have any additional effect on the cell depolarization. (d) The summary plot showing τ_decay_ of uI_GluT_ in the cells voltage‐clamped at −85, −65, and − 40 mV. (e) *Left*, sample traces of uI_GluT_ produced by glutamate uncaging while holding astrocyte at fixed V_m_ = −85 mV but with different [K^+^]_o_ = 2.5, 7.5, and 20 mM; *Right*, the summary plot showing normalized uI_GluT_ amplitude in the cell exposed to different [K^+^]_o_. (f) The summary plot showing τ_decay_ of uI_GluT_ in the cells voltage‐clamped at −85 mV and exposed to [K^+^]_o_ = 2.5, 7.5, and 20 mM. The data are presented as mean ± SEM. See the text for statistical test information

Our findings suggest that K^+^ efflux through postsynaptic NMDARs decreases glutamate uptake. The two candidate underlying mechanisms are (1) depolarization of the astrocytic membrane and (2) a decrease in the astrocytic transmembrane K^+^ gradient. Synaptically released glutamate rapidly binds to astrocytic transporters (Diamond & Jahr, 1997). From the bound state, glutamate^−^ can be translocated into the astrocyte cytoplasm, along with 3 Na^+^ and 1 H^+^, in exchange for 1 K^+^ (Grewer & Rauen, 2005). Therefore, the glutamate translocation step is both K^+^ − and voltage‐dependent (Grewer et al., 2008; Mennerick et al., 1999). To assess the relative contributions of K^+^ and voltage, we recorded I_GluT_ induced by the single‐pulse spot uncaging of glutamate that generates a typical single‐synapse (unitary) EPSC (uI_GluT_), as described previously (Henneberger et al., 2020), near the astrocyte soma, to minimize voltage‐clamp error (Figure 3a), under the two sets of conditions as follows. Firstly, we obtained the relationship between the extracellular K^+^ concentration and astrocyte membrane potential (Figure 3b). An increase in extracellular K^+^ produced similar astrocyte depolarization as previously reported (Ge & Duan, 2007). Second, we mimicked the K^+^‐induced depolarization by holding the cell in voltage‐clamp mode at the membrane potentials seen at high K^+^. The membrane depolarization alone reduced uI_GluT_ to the same degree as did the corresponding K^+^ concentration uI_GluT_ (*p*_K+_ = 0.18; *p*_depolarization_ < .001; interaction: *F*[2, 36] = 0.5, *p* = 0.59; *n =* 7; two‐way RM ANOVA; Figure 3c). These results suggest that depolarization alone can attenuate glutamate uptake, thus occluding the effects of K^+^ elevations. We also observed a depolarization‐dependent increase in τ_decay_ of uI_GluT_ (*F*[2, 21] = 6.2, *p* = .007, *n* = 8, one‐way RM ANOVA; Figure 3d). Correspondingly, when different extracellular K^+^ concentrations were applied under a constant membrane potential of −85 mV, no significant change in the amplitude of uI_GluT_ (*F*[2, 13] = 2.2, *p* = 0.14, *n* = 4; 8; 4, one‐way RM ANOVA) or in τ_decay_ (*F*[2, 19] = 7.6, *p* = .003, *n* = 11; 7; 4 for each condition one‐way RM ANOVA) was observed (Figure 3e, f). These findings suggest that physiologically relevant elevations in extracellular K^+^ affect I_GluT_ through astrocyte depolarization rather than by reducing the driving force for K^+^.

### 3.5 A biophysical underpinning of the relationship between extracellular K^+^ and glutamate uptake

To understand through which biophysical mechanism an extracellular K^+^ rise can affect the kinetics of astrocytic glutamate uptake, we employed a realistic, multi‐compartmental 3D model of a *stratum radiatum* astrocyte, which was developed and validated earlier using the NEURON‐compatible model builder ASTRO (Savtchenko et al., 2018). The model features known membrane astrocytic mechanisms, including GLT‐1 transporters and K_ir_4.1 K^+^ channels, distributed in the model membrane to match multiple experimental observations (Materials and Methods).

**FIGURE 4:**
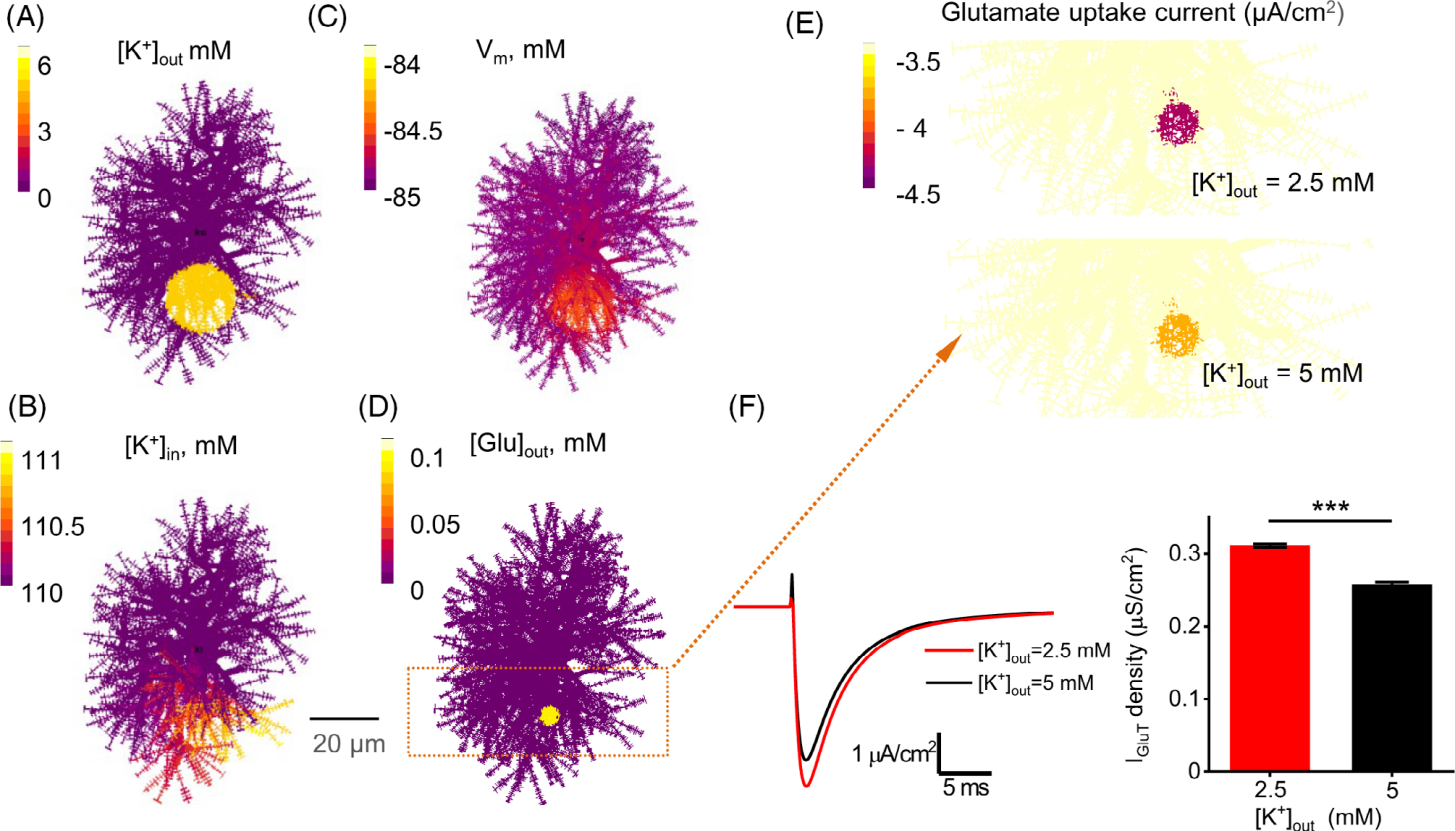
Realistic astrocyte model reveals the effect of extracellular K^+^ rises on local dynamics of astrocytic glutamate uptake. (a–c) Cell‐shape diagram illustrating a realistic 3D ASTRO‐NEURON‐based model of a CA1 astrocyte that incorporates known astrocytic membrane mechanisms including GLT‐1 transporters and K_ir_4.1 channels. Color‐coding provides snapshots (2D projections, 0.9 s postonset), showing how a local [K^+^]_out_ rising (from 2.5 to 5 mM over a 20 μm spherical area, 1 s duration) affects [K^+^]_in_ (c) and transmembrane voltage (A_3_). (d) Cell‐shape diagram illustrating ‘multi‐synaptic’ release of glutamate (0.1 mM for 1 ms, at 0.9 s postonset) within a small extracellular area (color hotspot, false color scale applies). (e) Landscape of glutamate uptake current density (1 ms postrelease, overall ROI shown as a dotted rectangle in [b]), under baseline [K^+^]_out_ = 2.5 mM (*top*) and during [K^+^]_out_ elevation to 5 mM as shown in (a–c). (f) *Left*, Glutamate uptake current kinetics over the affected cell area (color hotspot) shown in (c), at two [K^+^]_out_, as indicated. *Right*, Summary of a series of simulation tests recording I_GluT_ density at 10 different distances from the modeled cell soma (*n =* 10). The data are presented as mean ± SEM, ***p < .001, paired‐sample *t* test

First, we employed the model to simulate extracellular K^+^ elevation (from 2.5 to 5 mM over a 20 μm spherical area, 1 s duration) within the 3D astrocyte territory (Figure 4a). This generated local K^+^ influx through K_ir_4.1 channels, triggering diffuse equilibration of intracellular K^+^ across the tortuous cell lumen (Figure 4b). The evolving dynamics of extra‐ and intracellular K^+^ was paralleled by local membrane depolarization (Figure 4c). Aiming to mimic synchronous multi‐synaptic glutamate release, we also simulated a brief extracellular glutamate rise (0.1 mM for 1 ms, at 0.9 s postonset) within a 3 μm spherical area (Figure 4d) placed inside the area of extracellular K^+^ elevation. To understand the effect of extracellular K^+^ rises on glutamate uptake, we, therefore, ran the glutamate release test in two conditions, one at the baseline extracellular K^+^ and one during K^+^ elevation, as above. In these tests, cloud‐computing modeling could reveal 3D membrane profiles of transporter current at high (nanoscopic) resolution (Figure 4e). The I_GluT_ value volume‐averaged over the same glutamate uptake hotspot showed a significantly lower (by ~15%) amplitude during K^+^ elevation (Figure 4f). This decrease was similar to the effect of the K^+^ application of I_GluT_ induced by glutamate uncaging (Figure 3c). These simulation results provide a theoretical biophysical basis to the hypothesis that transient elevations of extracellular K^+^ could inhibit local glutamate uptake by astrocytes.

### 3.6 Synaptic K^+^ efflux boosts glutamate spillover

**FIGURE 5:**
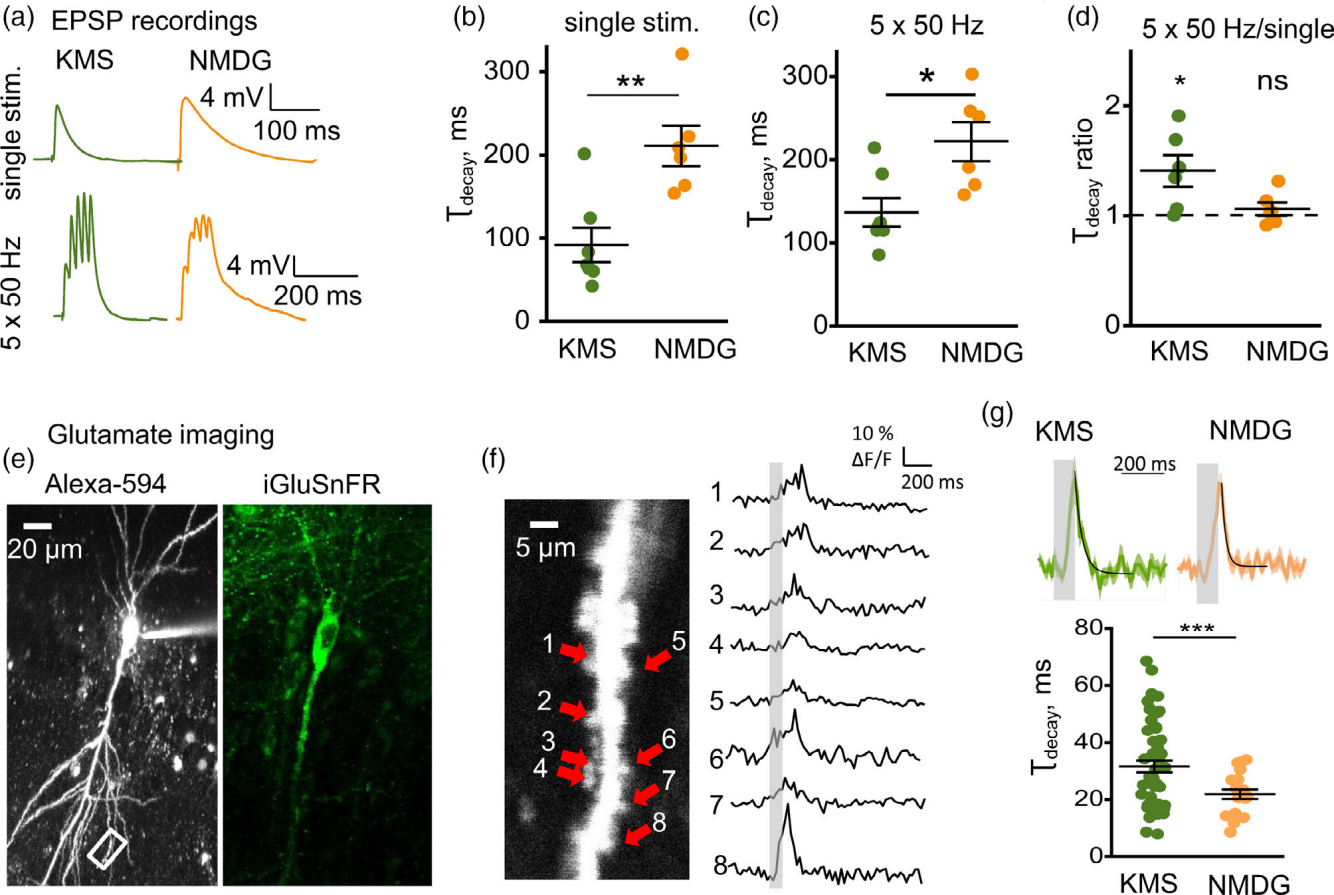
Synaptic K^+^ efflux increases glutamate dwell time in the synaptic cleft and enhances its spillover. (a) Sample traces of EPSPs recorded in CA1 pyramidal neurons filled with either KMS‐ or NMDG‐based internal solutions in response to single‐pulse or 5 × 50 Hz stimulation, as indicated. (b) and (c) The summary plot of τ_decay_ of EPSPs in response to a single‐pulse (b) and 5 × 50 Hz (C) stimulation. Replacement of intracellular K^+^ (green) for NMDG (orange) significantly increased τ_decay_ in both cases. (d) The summary plot showing the ratio of τ_decay_ of EPSP in response to 5 × 50 Hz stimulation to τ_decay_ of EPSP in response to single‐pulse stimulation. The activity‐dependent prolongation of EPSP was observed when the cell was filled with KMS‐ but not with an NMDG‐based solution. (e) CA1 pyramidal neuron expressing iGluSnFR loaded with fluorescent dye Alexa–594 via patch pipette, shown in two emission channels, as indicated. (f) *Left*, zoomed‐in area boxed in E; arrows, analyzed dendritic spines. *Right*, glutamate traces recorded at the corresponding spines. Gray bar ‐ stimulation. (g) Averaged traces and a summary plot showing the τ_decay_ of glutamate transients recorded from 53 dendritic spines (five cells) and 20 dendritic spines (three cells) with KMS and NMDG‐based solutions, respectively. The substitution of K^+^ with NMDG shortened the glutamate dwell time around the synapses. The data are presented as mean ± SEM. ns *p* > .05, *p < .05, **p < .01, and ***p < .001, two‐sample (b,c,g) and one‐sample (d) *t* test

Changes in I_GluT_ may not faithfully reflect glutamate concentration changes in the extracellular space as I_GluT_ reflects, in large part, the rate of glutamate translocation across the astrocytic membrane. Glutamate binding occurs when free transporters are in abundance. However, during repetitive stimuli, slowing down or reducing I_GluT_ would reflect a greater fraction of transporters bound by glutamate (before the transmembrane transfer step) on the astrocytic surface. As the fraction of free transporters on the astrocytic surface decreases, the probability of glutamate molecules traveling further from the release sites increases. In this case, I_GluT_ would reflect the dynamics of glutamate escape and removal to a much greater degree. When the glutamate translocation rate is reduced by astrocytic depolarization, more transporters can buffer extracellular glutamate (Diamond & Jahr, 1997). Such increased glutamate buffering (binding‐unbinding) by transporter molecules could increase the dwell time of glutamate near the synaptic cleft (Lehre & Rusakov, 2002; Zheng et al., 2008). Therefore, we attempted to assess the dynamics of extracellular glutamate concentration using the tests as follows. First, we recorded excitatory postsynaptic potentials (EPSPs) in CA1 pyramidal neurons in response to a single stimulus and to 5 × 50 Hz stimulation, in the presence of 100 μM picrotoxin, a GABA_A_ receptor antagonist (Figure 5a). The cut was made between CA1 and CA3 regions to prevent epileptiform activity. One of the two intracellular solutions was used: potassium methanesulfonate (KMS)‐based or N‐methyl‐D‐glucamine (NMDG)‐based. Having NMDG in the postsynaptic cell reduced the use‐dependent facilitation of EPSPs (Figure 5a), lending support to the earlier finding that K^+^ efflux boosts presynaptic release efficacy (Shih et al., 2013). In contrast to NMDG‐based solution, KMS‐based solution allowed K^+^ efflux through postsynaptic receptors, which curtailed EPSP. Therefore, the decay time constant (τ_decay_) of EPSP was smaller in KMS than in NMDG recordings (KMS: 91.6 ± 20.63 ms, *n =* 7; NMDG: 210.8 ± 24.5 ms, *n =* 6; *p =* .003, two‐sample *t*‐test; Figure 5b). The τ_decay_ of burst EPSP in response to 5 × 50 Hz stimulation was increased in KMS but not in NMDG (KMS: τ_decay_ = 136.52 ± 17.02 ms, τ_burst/single_ = 1.4 ± 0.14, *n =* 6, *p =* .03, single‐sample *t*‐test for the ratio; NMDG: τ_decay_ = 221.9 ± 23.5, τ_burst/single_ = 1.06 ± 0.05, *n =* 6, *p =* 0.34, single‐sample *t*‐test for the ratio; *p < .001*, two‐sample *t*‐test between ratios; Figure 5c, d).

This finding suggests that glutamate spillover is regulated by K^+^ efflux through postsynaptic receptors. Alternatively, this result may reflect the voltage and activity‐dependent regulation of K^+^ current that curtails EPSPs (Ichinose et al., 2003). Therefore, we next attempted to directly evaluate extracellular glutamate transient with genetically encoded glutamate sensor iGluSnFR (Figure 5e). CA1 pyramidal neurons expressing the sensor (methods as described earlier [Jensen et al., 2019; Jensen et al., 2017]) were loaded through the patch pipette with either KMS‐ or NMDG‐based intracellular solution. We documented synaptically evoked extracellular glutamate transients using 500 Hz line‐scans placed across visually identified dendritic spines in the second‐order apical dendrite branches (Figure 5f). Glutamate responses to burst stimulation (5 × 50 Hz) were thus recorded. The value of τ_decay_ for glutamate transients was significantly larger if the cell was loaded with the KMS‐based intracellular solution compared to the NMDG‐based solution (KMS: 31.56 ± 2.04 ms, *n =* 53 spines from five cells; NMDG: 21.8 ± 1.65 ms, *n =* 20 spines from 3 cells; *p <* .001, two‐sample *t* test; Figure 5g). This finding supports the suggestion that activity‐dependent prolongation of EPSPs is due to glutamate spillover boosted by K^+^ efflux from the postsynaptic terminal.

### 3.7 Synaptic K^+^ efflux boosts intersynaptic crosstalk

**FIGURE 6:**
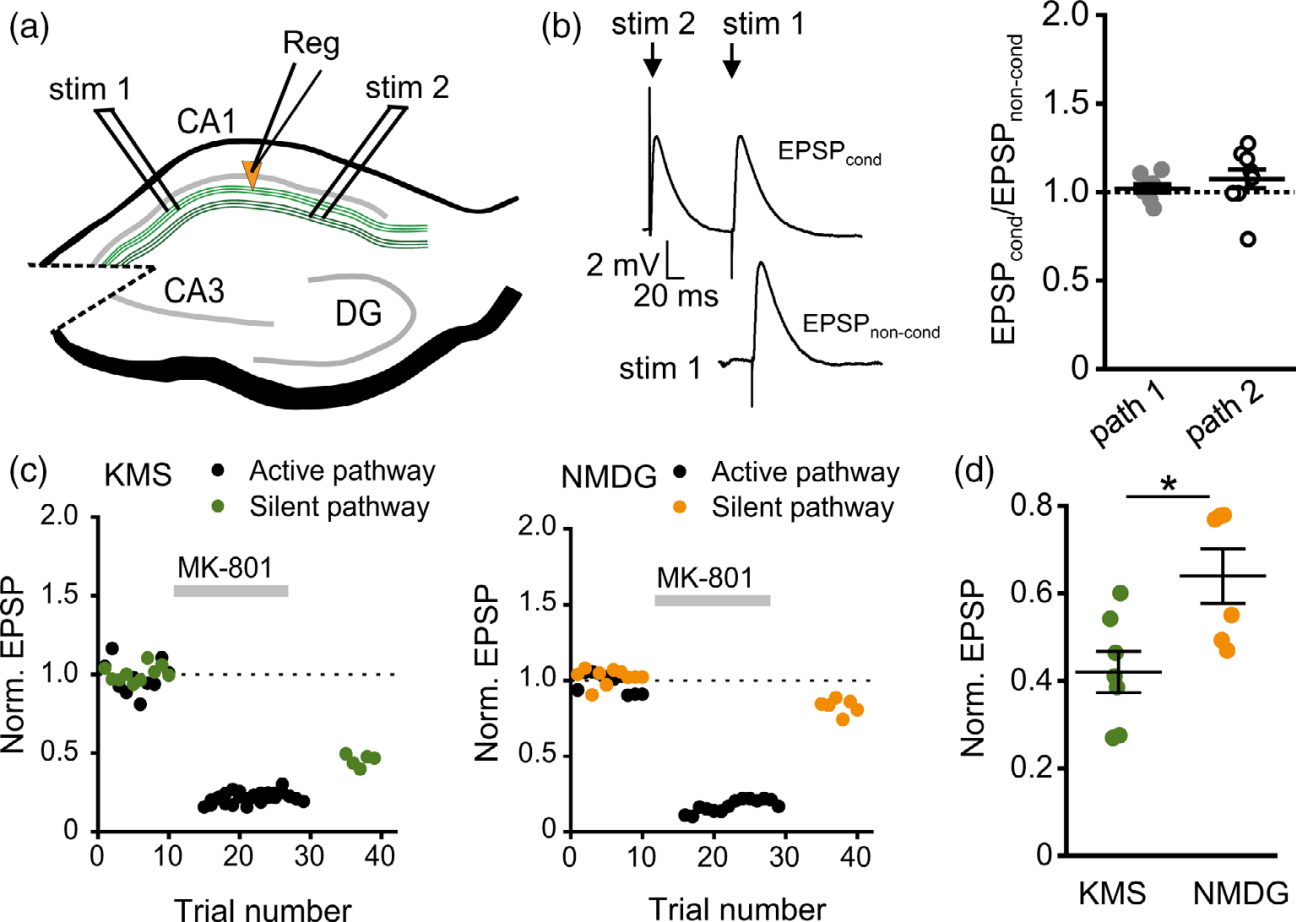
Postsynaptic K^+^ efflux enhances intersynaptic crosstalk. (a) A schematic showing the position of two stimulating (SC stim 1 and SC stim 2) and one recording (Reg) electrodes. Stimulating electrodes were placed to activate two parallel pathways of Schaffer collaterals. EPSPs were recorded from CA1 pyramidal neurons. (b) The test on pathway independence. *Left*, sample traces of conditioned EPSP (EPSP in one pathway [stim 1] was preceded by EPSP in the other pathway [stim 2]) and unconditioned EPSP in response to stim 1 only. *Right*, a ratio of conditioned EPSP (stim 1 after stim 2) versus nonconditioned EPSP (stim 1 only) in pathway 1 (path 1) and a ratio of conditioned EPSP (stim 2 after stim 1) versus nonconditioned EPSP (stim 2 only) in pathway 2 (path 2). (c) Examples of two pathway recordings with KMS‐(*left*) and NMDG‐based (*right*) internal solutions. Gray bar ‐ application of MK‐801, activity‐dependent channel blocker of NMDARs. Replacement of intracellular K^+^ for NMDG reduced the blockade of the silent pathway during stimulation of the active pathway. This points to reduced glutamate spillover. (d) The summary plot showing EPSP reduction in the silent pathway. The EPSPs were normalized to their baseline amplitude. The reduction was significantly larger when the postsynaptic cell was filled with K^+^. The data are presented as mean ± SEM. ns *p* > .05, *p < .05, two‐sample *t* test

Enhanced glutamate spillover can facilitate intersynaptic crosstalk via high‐affinity NMDARs. To test if synaptic K^+^ efflux promotes the crosstalk, we performed a modified two‐pathway experiment (Henneberger et al., 2020; Scimemi et al., 2004). Briefly, NMDARs‐mediated EPSPs in CA1 pyramidal neurons were pharmacologically isolated with NBQX and picrotoxin, AMPAR and GABA_A_ receptor blockers, and recorded during depolarizing voltage steps to −40 mV. Two bipolar electrodes were placed in CA1 *stratum radiatum* on the opposite sides of the slice to recruit independent afferent pathways of Schaffer collaterals (Figure 6a). The lack of cross‐facilitation of EPSPs in response to paired stimulation (first‐second and second‐first pathway) confirmed pathways independence (Scimemi et al., 2004) (Figure 6b). After baseline recordings of EPSPs in both pathways, the stimulation of one pathway was stopped (silent pathway), and 4 μm MK‐801, an activity‐dependent NMDARs blocker, was applied. High‐frequency stimulation (HFS) was delivered to the second pathway (active pathway). This stimulation led to the relatively rapid blockade of NMDARs at the active pathway synapses and some synapses at the silent pathway, which were reached by the glutamate escaping the active synapses (Figure 6c). The proportion of synapses blocked by MK‐801 at the silent pathway was estimated upon the drug washout and was significantly larger when the cell was filled with KMS‐ than NMDG‐based intracellular solution (EPSP: 42 ± 4% of baseline for KMS‐based solution, *n =* 7; 63.9 ± 6% of baseline for NMDG‐based solution, *n =* 6; *p =* .01, two‐sample *t*‐test; Figure 6c, d). This finding suggests that K^+^ efflux through postsynaptic receptors promotes glutamate spillover‐mediated intersynaptic crosstalk involving high‐affinity NMDARs.

## 4 DISCUSSION

In the course of excitatory synaptic transmission, astrocytes clear released glutamate and excess of K^+^ from the extracellular space (Verkhratsky & Nedergaard, 2018). We observed an activity‐dependent increase in the amplitude and τ_decay_ of synaptically‐induced I_GluT_ in hippocampal astrocytes. The increase of the amplitude of I_GluT_ is consistent with the reported facilitation of release probability mediated by K^+^ accumulation in the synaptic cleft (Contini et al., 2017; Shih et al., 2013). Indeed, this increase was suppressed by the blockade or genetic deletion of the postsynaptic of NMDARs, a major source of K^+^ efflux during synaptic transmission. Similarly, the increase of τ_decay_ was abolished in these tests. The result is consistent with the early report suggesting that glutamate transporters are not overwhelmed during HFS upon blockade of ionotropic postsynaptic receptors (Diamond & Jahr, 2000). Thus, glutamate transporters can effectively clear glutamate in hippocampal CA1 unless local glutamate translocation is partly suppressed by K^+^ efflux through postsynaptic NMDARs. Depolarization of perisynaptic astrocytic leaflets or a decrease of the transmembrane K^+^ gradient, or both, can potentially underpin reduced glutamate uptake (Grewer et al., 2008). However, we found that K^+^ elevation alone does not affect I_GluT_, while astrocyte depolarization increases its τ_decay_. How much K^+^ accumulation during synaptic transmission can depolarize leaflets cannot be directly measured because these processes are beyond optical resolution for light‐microscopy voltage imaging, nor can it be accessed with electrode‐based techniques. Previous simulation studies suggest that K^+^ can rise up to 5 mM in the synaptic cleft during a single EPSC (Shih et al., 2013). However, the K^+^ concentration drops rapidly outside of synaptic cleft, and its effect on the astrocyte may be strongly attenuated. Nevertheless, space and time‐averaged extracellular [K^+^]_o_ during HFS can increase by several millimoles (Durand et al., 2010). In such cases, the local perisynaptic K^+^ elevation should be several times higher. Although it was not technically feasible to probe the effects of K^+^ hotspots on the microscopic scale, we found that elevation of K^+^ to 7.5 mM depolarizes the astrocyte from −82.43 ± 0.79 mV to −63.8 ± 2.08 mV, which significantly increases τ_decay_ of uI_GluT_.

Whilst changes in I_GluT_ reflect glutamate translocation across the cell membrane, the extracellular glutamate concentration evolves on a much faster time scale. Reduced glutamate translocation rate may increase the dwell time hence the chances of glutamate unbinding extracellular transporter binding sites, which in turn could slow down (passively buffer) glutamate diffusion and prolong the glutamate transient tail (Rusakov, 2001). The relatively low membrane mobility of the main astroglial transporter GLT1 near synaptic sites (Al Awabdh et al., 2016; Michaluk et al., 2021; Murphy‐Royal et al., 2015) might contribute further to such phenomena. Indeed, imaging with glutamate sensor iGluSnFR revealed a decrease in τ_decay_ of glutamate transient when K^+^ in the postsynaptic cell was replaced with NMDG. Consistent with a longer glutamate dwell‐time, the activity‐dependent prolongation of EPSPs was observed only when the postsynaptic cell contained a physiological range concentration of K^+^, but not when K^+^ was replaced with NMDG. However, an increase in local glutamate dwell‐time does not reveal the efficiency of glutamate spillover. The latter may be enhanced with reduced glutamate uptake and reduced with increased glutamate buffering, or indeed under withdrawal of perisynaptic leaflets (Henneberger et al., 2020). We employed a two‐pathway experiment to demonstrate that glutamate spillover also depends on postsynaptic K^+^ efflux.

Our findings suggest that blockade of postsynaptic NMDARs reduces activity‐dependent facilitation of glutamate release and spillover onto neighboring sites. However, an alternative explanation is that blocking postsynaptic receptors in the entire slice suppresses recurrent excitation of CA3 pyramidal neurons and hence activity‐dependent recruitment of additional CA3 ‐> CA1 fibers. While this could explain the NMDAR‐dependent facilitation of I_GluT_, it could not explain the documented prolongation of I_GluT_ decay time. Moreover, the CA1‐GluN1‐KO mice showed reduced activity‐dependent facilitation and the reduced prolongation of I_GluT_. Replacing intracellular K^+^ for NMDG similarly reduced the activity‐dependent facilitation and prolongation of EPSPs in pyramidal neurons. Since CA3 pyramidal neurons have not been affected in either experiment, these results effectively rule out polysynaptic effects.

The source of K^+^ released by the postsynaptic neuron is another crucial issue. In addition to their own Ca^2+^ permeability, NMDARs are linked to Ca^2+^ dependent K^+^ channels that can potentially contribute to K^+^ efflux (Ngo‐Anh et al., 2005; Shah & Haylett, 2002; Zhang et al., 2018). However, extracellular Ca^2+^ removal does not reduce I_K_ in astrocytes induced by the activation of neuronal NMDARs with glutamate puff (Shih et al., 2013). Hence, a significant contribution of Ca^2+^‐dependent K^+^ channels to K^+^ efflux during synaptic transmission is unlikely. Another possibility is the activation of voltage‐gated K^+^ channels during EPSPs. However, blockade of NMDARs reduces field EPSPs to a much lesser extent than it reduces I_K_ (Shih et al., 2013). Thus, no major contribution of voltage‐gated K^+^ channels to K^+^ efflux during synaptic transmission has been observed.

Another question is to what extent K^+^ released at an individual synapse affects glutamate uptake in perisynaptic astrocytic leaflets. Biophysical models predict that K^+^ concentration rapidly drops with distance from the cleft, with no effect on astrocytic processes at nearest‐neighbor synapses (Shih et al., 2013). The low input resistance combined with a large membrane area severely limits the spread of current‐triggered membrane depolarization in astrocytes. Therefore, increasing the number of activated synapses should not affect the kinetics of glutamate uptake unless their K^+^ hotspots become overlapped in space. The latter scenario could occur during synchronous activation of multiple neighboring synapses or during epileptic bursts. Such events could produce wide‐spread elevations of extracellular K^+^ affecting large astrocyte territories.

In summary, we conclude that glutamate spillover is prevented by efficient glutamate uptake unless astroglial transporters are downregulated or withdrawn from the immediate synaptic environment. Indeed, early reports demonstrated that lowering recording temperature to decrease glutamate uptake efficiently promotes glutamate spillover (Asztely et al., 1997; Kullmann & Asztely, 1998). Also, early findings highlighted the role of NMDARs to detect spillover while attributing this fact to higher glutamate affinity of these receptors (Diamond, 2001; Rusakov & Kullmann, 1998).

Our findings indicate that K^+^ efflux through postsynaptic NMDARs depolarizes astrocytic leaflets and thus reduces local glutamate uptake. What could be the physiological role of this phenomenon? In hippocampal circuitry, enhanced glutamate spillover has been associated with co‐operative action of dendritic NMDARs, such as receptor ‘priming’ (Arnth‐Jensen et al., 2002; Hires et al., 2008). It has been found to underlie functional inter‐synaptic crosstalk (Arnth‐Jensen et al., 2002; Lozovaya et al., 1999; Scimemi et al., 2004), also contributing significantly to heterosynaptic plasticity (Vogt & Nicoll, 1999), and activation of metabotropic glutamate receptors outside the synaptic cleft (Min et al., 1998; Semyanov & Kullmann, 2000).

Recent reports suggest that a decrease in glutamate uptake can shift the sign of synaptic plasticity, reducing long‐term potentiation (LTP) and promote long‐term depression (LTD) (Valtcheva & Venance, 2019). Rate‐based LTP induction in one set of synapses requires NMDARs activation and thus should lead to K^+^ hotspots in the perisynaptic space. This K^+^ should, in turn, depolarize astrocytic leaflets and downregulate glutamate uptake. However, leaflets within brain active milieu are ‘shared’ between neighboring synapses (Gavrilov et al., 2018; A. Semyanov & Verkhratsky, 2021). We thus speculate that LTP in one set of synapses could suppress LTP and facilitate LTD in their neighbors if they are activated immediately after while astrocyte is depolarized.

LTP induction per se could change the synapse's ability to release K^+^ into the extrasynaptic space (Ge & Duan, 2007). The number of synaptic AMPARs is thought to increase during LTP, and, although they should not contribute significantly to K^+^ efflux due to their fast inactivation, they can facilitate activation of NMDARs by removing their voltage‐dependent Mg^2+^ block (Shih et al., 2013). Therefore, LTP not only increases the quantal efficiency of the synapse but promotes K^+^‐dependent facilitation of glutamate release and spillover at this synapse, potentially complementing the effect of the (possibly transient) perisynaptic astrocytic leaflet withdrawal after LTP induction (Henneberger et al., 2020). K^+^‐dependent facilitation of presynaptic glutamate release after LTP could be also linked to the putative perisynaptic mechanism of LTP (Kullmann, 2012).

Our observations emphasize the physiological importance of changes in ionic concentrations in the synaptic cleft and perisynaptic space. Since the volumes of these spaces are tiny, the concentration changes could be significant. Accumulation of K^+^ in the synaptic cleft is paralleled by local Ca^2+^ depletion (Rusakov & Fine, 2003), which is also sensed by astrocytes (Torres et al., 2012) and might affect release efficacy in the opposite direction to that of excess K^+^. How the astrocyte mediated K^+^ buffering affects the time‐course of perisynaptic K^+^ elevation and how far K^+^ can diffuse in the extracellular space remains to be established.

## AUTHOR CONTRIBUTIONS

O.T. carried out electrophysiological and imaging experiments; P‐Y. S. carried out electrophysiological and glutamate uncaging experiments; Y.D. carried out electrophysiological experiments; L.P.S designed and carried out biophysical simulations; T.J.M. created transgenic animals; D.A.R. contributed to experimental and theoretical designs, data analysis; A.S. project idea and supervision, experimental design, data analysis, manuscript writing. All authors contributed to manuscript writing.

## CONFLICT OF INTEREST

The authors have no conflict of interest to declare.

## Supporting information


**Figure S1** Astrocytic K^+^ current scales with increases in stimulus strength but shows the unchanged use‐dependent increase during bursts.
A. Trace of characteristic *I*_*K*_ *+ I*_*GluT*_ current in response to a single stimulus applied to Shaffer collaterals; pink bar (200 ms after the last peak): current measurement window. Graph: statistical summary showing *I*_*K*_ during weak and strong stimuli. B. Experiments as in (A), but with a 5 × 50 Hz stimulus burst; notation as in (A). C. The summary graphs showing an *I*_*K*_ increase during 5 × 50 Hz bursts relative to the single stimulus *I*_*k*_.
The data are presented as mean ± SEM. ns *p* > .05 and *p < .05, two‐sample t‐test.

## Data Availability

The data that support the findings of this study are available from the corresponding authors upon reasonable request.
