# Supplementary material for: K+ efflux through postsynaptic NMDA receptors suppresses local astrocytic glutamate uptake
Source: Glia. 2022 Jan 27;70(5):961–74. doi: 10.1002/glia.24150 (PMC9132042; doi:10.1002/glia.24150)
Supplement: Supplementary file 1 — Figure S1 Astrocytic K+ current scales with increases in stimulus strength but shows the unchanged use‐dependent increase during bursts. A. Trace of characteristic I K + I GluT current in response to a single stimulus applied to Shaffer collaterals; pink bar (200 ms after the last peak): current measurement window. Graph: statistical summary showing I K during weak and strong stimuli. B. Experiments as in (A), but with a 5 × 50 Hz stimulus burst; notation as in (A). C. The summary graphs showing an I K increase during 5 × 50 Hz bursts relative to the single stimulus I k . The data are presented as mean ± SEM. ns p > .05 and *p < .05, two‐sample t‐test. [file GLIA-70-961-s001.doc]

Supplementary figure


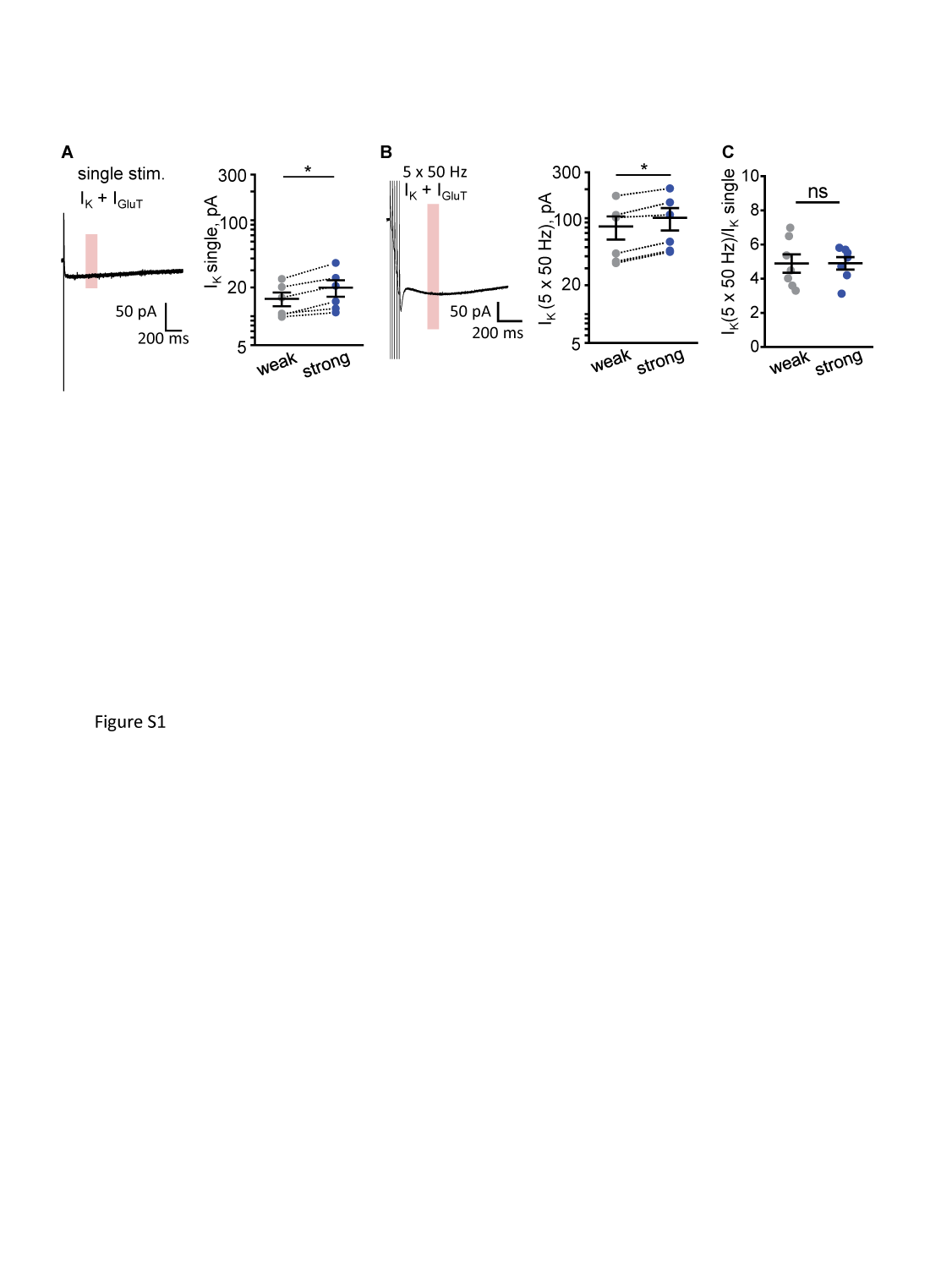


Figure S1. Astrocytic K^+^ current scales with increases in stimulus strength but shows the unchanged use-dependent increase during bursts.

A. Trace of characteristic *I_K_+I_GluT_* current in response to a single stimulus applied to Shaffer collaterals; pink bar (200 ms after the last peak): current measurement window. Graph: statistical summary showing *I_K_* during weak and strong stimuli. B. Experiments as in (A), but with a 5 x 50 Hz stimulus burst; notation as in (A). C. The summary graphs showing an *I_K_* increase during 5 x 50 Hz bursts relative to the single stimulus *I_k_*.

The data are presented as mean ± SEM. ns p > 0.05 and *p < 0.05, two-sample t-test.
